# Anterior Segment Ischemia after Strabismus Surgery

**DOI:** 10.4274/tjo.93824

**Published:** 2017-01-17

**Authors:** Emine Seyhan Göçmen, Yonca Atalay, Özlem Evren Kemer, Hikmet Yavuz Sarıkatipoğlu

**Affiliations:** 1 Ankara Numune Training and Research Hospital, Ophthalmology Clinic, Ankara, Turkey

**Keywords:** Anterior segment ischemia, Foster, sixth nerve palsy, transposition surgery

## Abstract

A 46-year-old male patient was referred to our clinic with complaints of diplopia and esotropia in his right eye that developed after a car accident. The patient had right esotropia in primary position and abduction of the right eye was totally limited. Primary deviation was over 40 prism diopters at near and distance. The patient was diagnosed with sixth nerve palsy and 18 months after trauma, he underwent right medial rectus muscle recession. Ten months after the first operation, full-thickness tendon transposition of the superior and inferior rectus muscles (with Foster suture) was performed. On the first postoperative day, slit-lamp examination revealed corneal edema, 3+ cells in the anterior chamber and an irregular pupil. According to these findings, the diagnosis was anterior segment ischemia. Treatment with 0.1/5 mL topical dexamethasone drops (16 times/day), cyclopentolate hydrochloride drops (3 times/day) and 20 mg oral fluocortolone (3 times/day) was initiated. After 1 week of treatment, corneal edema regressed and the anterior chamber was clean. Topical and systemic steroid treatment was gradually discontinued. At postoperative 1 month, the patient was orthophoric and there were no pathologic symptoms besides the irregular pupil. Anterior segment ischemia is one of the most serious complications of strabismus surgery. Despite the fact that in most cases the only remaining sequel is an irregular pupil, serious circulation deficits could lead to phthisis bulbi. Clinical properties of anterior segment ischemia should be well recognized and in especially risky cases, preventative measures should be taken.

## INTRODUCTION

Anterior segment ischemia is a rare but well documented complications of strabismus surgery. It generally manifests within 24 hours of surgery with blurred vision, lid and corneal edema, anterior segment cells, and hypotony.^1^ Advanced age, procedures involving multiple muscles, procedures on vertical muscles, hyperviscosity, and systemic vascular diseases are among the risk factors for anterior segment ischemia.^2^ In order to prevent this possible sight-threatening complication, surgical procedures which spare the anterior ciliary artery should be favored, especially in patients with risk factors.^3^ In this report, we discuss the precipitating factors, clinical features, and management of a case of anterior segment ischemia following full-thickness tendon transposition (with Foster suture).

## CASE REPORT

**Figure 1:**
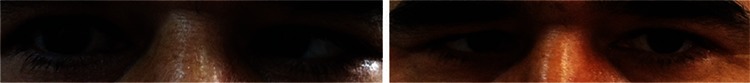
Right esotropia is evident preoperatively in primary position. Abduction is -4 limited

**Figure 2:**
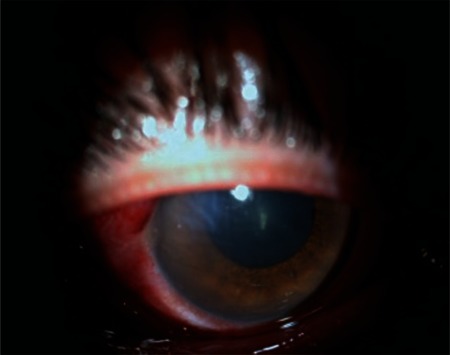
On postoperative day 1, corneal edema, Descemet membrane folds, irregular pupil and 3+ anterior segment cells are evident

**Figure 3:**
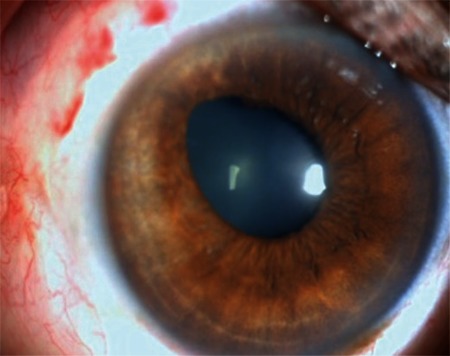
At postoperative 1 month, there are no pathologic signs other than pupil irregularity

**Figure 4:**
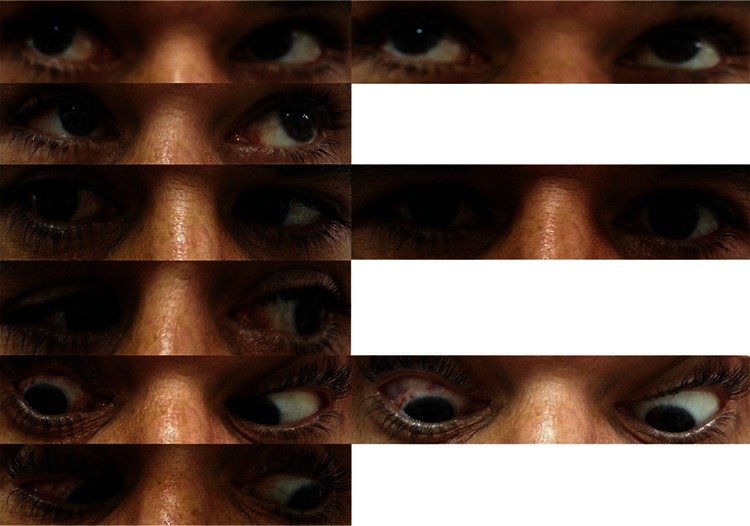
At postoperative 1 month, the patient is orthophoric in primary position. Abduction is -1 limited.

A 46-year-old male patient presented to our clinic with an approximately 18-month history of esotropia in his right eye and diplopia. The patient had no systemic diseases and it was learned that his symptoms developed following a car accident. The patient’s visual acuity was measured by Snellen chart as 20/20 in both eyes with refractive correction of -1.50 -1.00x95 on the right and -1.75 -0.50x120 on the left. Anterior and posterior examinations were normal. The patient had esotropia of 40 prism diopters and right eye abduction was graded as -4 (completely limited). Cranial tomography conducted 6 months earlier had revealed no pathology. Based on the findings, the patient was diagnosed with sixth nerve palsy and 18 months after the trauma he underwent 6 mm recession of the right medial rectus muscle. In postoperative follow-up, the patient’s esotropia in primary position continued and right eye abduction remained -3 limited. Ten months after the initial surgery, he underwent a full-thickness transposition of the superior and inferior rectus muscles to the lateral rectus muscle and a 5/0 multifilament nonabsorbable lateral fixation suture (Foster) was placed in the sclera 8 mm posterior to the lateral rectus insertion incorporating the superior rectus and one fourth of the lateral rectus muscle. Another 5/0 multifilament nonabsorbable lateral fixation suture was placed in the sclera 8 mm posterior to the lateral rectus insertion incorporating the inferior rectus and one fourth of the lateral rectus muscle. On postoperative day 1, biomicroscopic examination revealed corneal edema, Descemet’s membrane folds, mild hypotony, 3+ cells in the anterior chamber, and irregular mid-dilated pupil. The lens was not cataractous and fundus examination was normal. Visual acuity had declined to 20/40 on Snellen chart. Based on the findings, the diagnosis was anterior segment ischemia. Treatment with 0.1/5 mL topical dexamethasone drops (16 times/day), cyclopentolate hydrochloride drops (3 times/day) and 20 mg oral fluocortolone (3 times/day) was initiated the same day. After 1 week of treatment, the corneal edema had regressed and the anterior chamber was free of cells, but the pupil irregularity persisted. Visual acuity had improved to 20/28. Intraocular pressure was normal. The oral fluocortolone and topical dexamethasone were gradually discontinued over the course of 1 month. The patient’s visual acuity improved to 20/20, diplopia completely resolved and at postoperative 1 month, there were no remaining pathologic signs other than pupil irregularity. The patient was orthophoric in primary position and there was -1 limitation in right eye movement (Figures 1, 2, 3 and 4).

## DISCUSSION

Circulation to the anterior segment is provided by seven anterior ciliary arteries and two posterior ciliary arteries. One anterior ciliary artery supplies the lateral rectus muscle and two supply each of the other extraocular muscles.^1^ The vertical rectus muscle in particular has a major impact on anterior segment circulation.^2^ Various authors have reported anterior segment ischemia due to damage to this vascular network during strabismus surgery at rates ranging from 1/13,000 to 1/30,000.^3^ Anterior segment ischemia occurs as a result of interruption to the blood supply to the anterior segment following strabismus surgery. Permanent detachment of the rectus muscles cuts blood flow in the anterior ciliary arteries. Intravascular coagulative hematologic abnormalities and local or systemic factors impairing ocular circulation may also contribute to reduced blood flow.^4^ Advanced age, systemic vascular disease, hyperviscosity, diabetes mellitus, dysthyroid ophthalmopathy, and 360° scleral buckling surgery due to retinal detachment are among the risk factors associated with this complication.^5^ Anterior segment ischemia has been reported as a result of strabismus surgery in patients with a history of radiotherapy to treat tumors of the head and neck.^6^ As a general rule, including more than three rectus muscles in a single operation and performing a second rectus muscle surgery within six months of a previous rectus muscle surgery substantially increase the risk of anterior segment ischemia.^2^ Olver and Lee^7^ graded anterior segment ischemia as follows: Grade I: decreased iris perfusion; Grade II +pupil signs; Grade III: +uveitis; and Grade IV: +keratopathy. Although most patients’ iris circulation returns to baseline levels within 2 weeks after surgery, the recovery can last up to 12 weeks in some cases. Grade IV anterior segment ischemia in particular can lead to permanent vision loss due to cataract, corneal scar and macular changes. The condition is marked by blurred vision and edema of the eyelids, conjunctiva and cornea which usually appear the first day after strabismus surgery. The pupil is often mid-dilated and light reaction is weak. There may be a high concentration of cells in the anterior chamber, but intraocular pressure is low due to reduced circulation.^8^ Anterior segment angiography reveals diffuse iris leakage in acute-onset ischemia, versus pupil margin leakage and nodule-like vascular dilations in gradual-onset ischemia. In ischemias that cause iris atrophy, the areas of ischemia have distinct margins.^9^ Arterial circulation often recovers in the long term but in some patients, iris atrophy and pupil irregularity may persist.^1^ Kaeser and Klainguti^10^ noted relative iris ischemia in 4 of 10 patients that had previously undergone horizontal rectus muscle.

It is generally recommended to avoid procedures involving more than three rectus muscles in order to prevent anterior segment ischemia.^8^ Girard and Beltranena^11^ reported mild anterior segment necrosis due to impaired anterior ciliary artery circulation after tenotomy of three or more rectus muscles. In an experimental study on monkey eyes by Virdi and Hayreh,^12^ it was determined that simultaneous recession of two or three rectus muscles can cause mild to moderate anterior segment ischemia, whereas procedures involving four muscles can lead to serious, permanent changes. Another surgical method developed to prevent anterior segment ischemia and which is currently especially used in paralytic strabismus surgery is the Hummelscheim procedure. In this technique, the muscle fibers attached to the lateral halves of the superior and inferior rectus tendons are fixated to the lateral rectus tendon. Many surgeons prefer this technique because it preserves the vasculature.^13^ In 2001, Brooks et al.^14^ proposed an adapted version of the Hummelsheim procedure in which the vertical rectus muscle is resected 4-5 mm prior to transposition (augmented Hummelsheim procedure). Couser et al.^15^ performed medial rectus recession with the augmented Hummelsheim procedure in 9 patients and reported achieving orthophoria in primary position and improved abduction. None of their patients developed anterior segment ischemia. Klainguti et al.^16^ performed posterior fixation of the contralateral medial rectus in addition to the Hummelsheim procedure in 2 patients with sixth nerve palsy and reported favorable results with this combination. Rectus muscle plication reduces the likelihood of ischemia in the lost muscle and anterior segment.^17^ Oltra et al.^18^ claimed that plication surgery is safe in patients at high risk of developing postoperative anterior segment ischemia and demonstrated that patients who underwent plication developed fewer filling defects on iris angiography. Vijayalakshmi et al.^19^ performed left medial rectus muscle recession and vertical rectus muscle transposition to the lateral rectus augmented by lateral fixation sutures in a patient with sixth nerve palsy. The patient developed anterior segment ischemia which resolved upon the removal of the lateral fixation sutures and administration of medical treatment. Risk of anterior segment ischemia is also markedly higher within the first six months after rectus muscle surgery. However, there are documented cases of patients developing anterior segment ischemia years after initial surgery.^20^ Although surgeries that conserve the anterior ciliary arteries have been shown to reduce the risk of anterior segment ischemia, circulation may not always continue intra- and postoperatively in vessels believed to be saved. Ischemic complications may arise even after successful microvascular dissection and conservation. Cases have also been documented of patients developing anterior segment ischemia after vessel-sparing procedures.^21^ Some authors argue that conjunctival incisions made at the fornix spare the conjunctiva-Tenon’s capsule junction and are therefore less likely to cause ischemia than incisions made at the limbus.^22^ The Jensen procedure (muscle joining technique) is known to preserve ciliary circulation to some degree, but it is not a definitive solution.^23^ The minimally invasive strabismus surgery (MISS) technique described by Mojon^24^ reduces the risk of anterior segment ischemia by sparing the perilimbal episcleral vessels. Nevertheless, the use of local anesthetics containing epinephrine can still lead to ischemia.^25^ In patients at risk of anterior segment ischemia, intraocular pressure should be controlled preoperatively and local anesthesia without sympathomimetic activity should be used. Intraoperatively, peritomy of the conjunctiva should kept to a minimum, the rectus muscles should not be pulled excessively, and the long posterior ciliary arteries should be avoided.^5^

In the event of severe anterior segment ischemia, topical and systemic steroid therapy is used to suppress inflammation. Cycloplegic drugs can be used to prevent synechia; topical mannitol and 0.9% NaCl drops can be used to reduce corneal edema. Intraocular pressure should be controlled.^3^

In the present case, the patient could have been initially treated with a Botox injection to the medial rectus muscle and, considering the patient’s age and the possibility that a procedure involving multiple rectus muscles would be required, the surgery initially performed could have been reserved as a secondary measure. Furthermore, evaluation of the patient by iris fluorescein angiography may have provided a warning of the possibility of anterior segment ischemia.

## CONCLUSION

Anterior segment ischemia is one of the serious complications of strabismus surgery. Even though an irregular pupil is the only remaining sequel for many patients, serious circulation deficits can lead to outcomes as severe as phthisis bulbi. Ophthalmologists should be very familiar with the clinical features of anterior segment ischemia and take preventative measures in risky cases.

### Ethics

Peer-review: Externally peer-reviewed.
